# A trimeric glycosylated GH45 cellulase from the red abalone (*Haliotis rufescens*) exhibits endo and exoactivity

**DOI:** 10.1371/journal.pone.0301604

**Published:** 2024-04-18

**Authors:** L. Joshua Hernández-Benítez, Miguel A. Ramírez-Rodríguez, Alejandra Hernández-Santoyo, Adela Rodríguez-Romero

**Affiliations:** Instituto de Química, Universidad Nacional Autónoma de México, Ciudad de México, México; University of Kalyani, INDIA

## Abstract

The red abalone (*Haliotis rufescens*) represents North America’s most important aquaculture species. Its hepatopancreas is rich in cellulases and other polysaccharide-degrading enzymes, which provide it the remarkable ability to digest cellulose-rich macroalgae; nevertheless, its cellulolytic systems are poorly explored. This manuscript describes some functional and structural properties of an endogenous trimeric glycosylated endoglucanase from *H*. *rufescens*. The purified enzyme showed a molecular mass of 23.4 kDa determined by MALDI-TOF mass spectrometry, which behaved as a homotrimer in gel filtration chromatography and zymograms. According to the periodic acid-Schiff reagent staining, detecting sugar moieties in SDS-PAGE gel confirmed that abalone cellulase is a glycoprotein. Hydrolysis of cello-oligosaccharides and *p*-nitrophenyl-*β*-D-glucopyranosides confirmed its endo/exoactivity. A maximum enzyme activity toward 0.5% (*w*/*v*) carboxymethylcellulose of 53.9 ± 1.0 U/mg was achieved at 45°C and pH 6.0. We elucidated the abalone cellulase primary structure using proteases and mass spectrometry methods. Based on these results and using a bioinformatic approach, we identified the gene encoding this enzyme and deduced its full-length amino acid sequence; the mature protein comprised 177 residues with a calculated molecular mass of 19.1 kDa and, according to sequence similarity, it was classified into the glycosyl-hydrolase family 45 subfamily B. An AlphaFold theoretical model and docking simulations with cellopentaose confirmed that abalone cellulase is a *β*-sheet rich protein, as also observed by circular dichroism experiments, with conserved catalytic residues: Asp26, Asn109, and Asp134. Interestingly, the AlphaFold-Multimer analysis indicated a trimeric assembly for abalone cellulase, which supported our experimental findings. The discovery and characterization of these enzymes may contribute to developing efficient cellulose bioconversion processes for biofuels and sustainable bioproducts.

## Introduction

The increasing interest in seaweeds as biomass sources for bioethanol production has heightened the need for cheaper and more sophisticated cellulases capable of completely hydrolyzing the *β*-1,4-bonds between *β*-D-glucose residues in cellulose. The effective hydrolysis of this polysaccharide requires a collection of three enzymes: endoglucanases, cellobiohydrolases or exoglucanases, and *β*-glucosidases, which act sequentially and synergistically [1]. Based on amino acid sequence and structure similarities [2], the CAZy database (http://www.cazy.org/) has classified endoglucanases into fifteen glycosyl-hydrolase families (GHs 5–10, 12, 26, 44, 45, 48, 51, 74, 124, 148). The most well-studied cellulases are often composed of a single protein chain that contains a catalytic domain and carbohydrate-binding module, which are responsible for cellulose degradation. However, it has also been reported that the association or quaternary structure of some carbohydrate hydrolases, like the trimeric glycosyl-hydrolase family 5 (GH5) endoglucanase from the marine bacterium *Saccharophagus degradans*, is essential for enzyme activity [3]. In particular, endoglucanases belonging to the glycosyl-hydrolase family 45 (GH45) are smaller than other GHs and exhibit broad substrate specificity [4]. Their structural characteristic is a six-stranded double-*ψ β*-barrel core surrounded by loops and *α*-helices. The amino acid residues involved in catalysis are two aspartic acids and an asparagine [5]. Based on phylogenetics, GH45 enzymes are classified into three subfamilies: A, B, and C. Cellulases belonging to subfamily B have been detected in mollusks; nonetheless, their structural and functional properties are still obscure [6, 7].

Although fungal and bacterial cellulases have been studied in detail and used for industrial and biotechnological purposes, few investigations on the properties of cellulases from animals have been reported. Interest in metazoan cellulases before 1998 was scarce since they were considered the products of symbiotic microorganisms in the host’s digestive tract [8]. The discovery of a cellulase gene in the termite *Reticulitermes speratus* [9] overturned the long-believed theory of the origin of these enzymes in animals; since then, endogenous cellulases have been isolated from terrestrial and marine herbivorous invertebrates [10]. However, few researchers focused on studying native cellulases from abalones [11–13]. The red abalone (*Haliotis rufescens* Swainson, 1822) represents North America’s most important aquaculture species; nonetheless, during its industrial canning processing the digestive gland, or hepatopancreas, is removed and discarded without any effort for recovery. The abalone hepatopancreas, which is rich in cellulases and other polysaccharide-degrading enzymes, is an important organ involved in defense functions, integration of metabolism, and immunity; therefore, abalone proteins have evolved with unique properties and functionalities [14]. Fish and shellfish waste products, such as viscera and head, are excellent sources of proteins and other value-added biomolecules. As marine metazoans have adapted to extreme and changing environmental conditions, enzymes purified from these organisms may exhibit advantages over their terrestrial orthologues, providing them with potential applications in many fields [15, 16].

In this study, we isolated an endoglucanase from the hepatopancreas of the red abalone, analyzed its functional and structural properties, and named it HrGH45. This biomolecule represents the first endogenous glycosylated cellulase from abalone that exhibits enzyme activity in its trimeric form. HrGH45 comprised only the catalytic domain and showed interesting enzymatic properties. Our structural analysis allowed its classification into the GH45 subfamily B. We identified the gene encoding HrGH45; thus, our findings will provide the basis for this enzyme’s over-expression. The exploration of glycosyl-hydrolases in abalone and other marine organisms provides exciting opportunities for bioprospecting and the discovery of novel enzymes with valuable applications.

## Materials and methods

### Materials

Living abalones identified as *H*. *rufescens* were collected on the coast of Ensenada, Baja California, México. They were transported to the laboratory and stored at -50°C until used. All reagents and solvents were of analytical, biochemical, or chromatographic grade, purchased from various commercial suppliers, and used without further purification. No specific permits were required for the described investigation, as the red abalone we used comes from a farm dedicated to producing this species for human consumption. Besides, it is a common marine invertebrate species in North America and is not endangered or protected.

### Purification of HrGH45

All purification steps were performed at 4°C unless otherwise indicated. The hepatopancreas (approximately 14 g) of one abalone was mechanically homogenized using an immersion blender (Oster FPSTHB2610W) with 200 mL of 50 mM sodium acetate buffer pH 6.0 containing 200 mM NaCl, 1 mM phenylmethylsulfonyl fluoride (PMSF), 1 mM EDTA, and 0.05% (*w*/*v*) NaN_3_. The homogenate was centrifugated at 3,500 rpm for 60 min (Hermle Z 300 K) to remove tissue debris and precipitated proteins. Proteins in the supernatant were precipitated using a two-step fractionation with (NH_4_)_2_SO_4_ at 30 and 60% saturation. The precipitated formed at 30–60% saturation was collected by centrifugation at 14,000 rpm for 20 min (Beckman Coulter Avanti J-30I), resuspended in a minimum volume of 50 mM sodium acetate buffer pH 6.0 containing 200 mM NaCl, 1 mM EDTA, and 0.05% (*w*/*v*) NaN_3_ (buffer A), and dialyzed against the same solution at 4°C for 48 h. Dialysis was performed with two membranes (Spectrum Spectra/Por 3,500 Da MWCO) since cellulase action caused the inner bag’s puncturing. The dialyzed enzyme solution was filtered (Millipore, Durapore PVDF Membrane Filter, Ø 0.45 μm) and applied to a system consisting of two 8.8 O.D. × 145 mm ion exchange columns connected in series and packed with DEAE-Sepharose Fast Flow (Pharmacia Biotech) and CM-Sepharose Fast Flow (Sigma-Aldrich). The column system was equilibrated with buffer A and washed exhaustively with the same solution until the non-adsorbed proteins passed through it. The adsorbed proteins were eluted separately with a linear gradient of 0.2 M to 1.2 M NaCl in buffer A. Chromatography was developed at a 12 mL/h flow rate. Proteins that passed through the system contained cellulase activity; thus, they were pooled. The pooled fraction was filtered (Millipore, Durapore PVDF Membrane Filter, Ø 0.22 μm) and loaded onto an Agilent Bio SEC-3 HPLC column (3 μm, 150 Å, 7.8 I.D. × 150 mm, Agilent Technologies) installed in an Agilent 1100 HPLC instrument (Agilent Technologies). Chromatography was carried out at a 1 mL/min flow rate using buffer A as eluent. The fraction with cellulase activity was collected, concentrated by ultrafiltration (Millipore, Amicon Ultra-15 Centrifugal Filter, 3,000 MWCO), and re-chromatographed once under the same conditions. This fraction, named HrGH45, was used for further experiments.

The protein concentration at each purification step was determined by the bicinchoninic acid (BCA) method [17] with the Pierce BCA Protein Assay Kit (Thermo-Fisher Scientific) and bovine serum albumin (BSA) as a standard. We employed the enhanced protocol, covering a working range of 5–250 μg/mL. Briefly, 100 μL of standard or sample solution was added to 2 mL of working reagent solution, which was prepared by mixing 50 mL of BCA Reagent A and 1 mL of BCA Reagent B. The samples were covered and incubated at 60°C for 30 min, cooled to room temperature, and analyzed spectrophotometrically (Shimadzu UV-1900) by measuring the absorbance at 562 nm. Assays were performed in triplicate.

The purity of HrGH45 was verified using SDS-PAGE, which was carried out by the method of Laemmli [18] using a homogeneous 12% (*w*/*v*) polyacrylamide resolving gel in a Mini-PROTEAN system (Bio-Rad). After electrophoresis, protein bands were detected by staining with Coomassie brilliant blue R-250. Molecular masses were estimated using the Precision Plus Protein Unstained Standards (Bio-Rad). We confirmed the homogeneity of HrGH45 using C_18_-reversed-phase high-performance liquid chromatography. An enzyme solution (0.1 mg/mL in buffer A) was filtered (Millipore, Durapore PVDF Membrane Filter, Ø 0.22 μm) and applied to an Agilent Zorbax 300SB-C18 HPLC column (5 μm, 4.6 I.D. × 150 mm, Agilent Technologies) installed in an Agilent 1100 HPLC instrument (Agilent Technologies). The mobile phase, consisting of 0.1% (*v*/*v*) trifluoroacetic acid (TFA) in Milli-Q water (solvent A) and 0.12% (*v*/*v*) TFA in acetonitrile (ACN, solvent B), was delivered at a 1 mL/min flow rate. Gradient elution was performed as follows: 0 min, 0% solvent B; 5 min, 0% solvent B; 6 min, 44% solvent B; 15 min, 44.5% solvent B; 16 min, 100% solvent B; 18 min, 100% solvent B. The mobile phase was freshly prepared. Detection was set at 280 nm and the experiment was performed at 25°C.

### Enzyme assays: Cellulase activity, specificity, and mode of action

Cellulase activity was determined by measuring the amount of reducing sugars released from the hydrolysis of carboxymethylcellulose sodium salt low viscosity (CMC, Sigma-Aldrich) using the 3,5-dinitro salicylic acid (DNS, Sigma-Aldrich) method [19]. The reaction mixture consisted of 50 μL of HrGH45 (0.01–0.1 mg/mL) in buffer A, 200 μL of buffer A, and 250 μL of 1% (*w*/*v*) CMC dissolved in the same buffer. After incubation at 45°C for 30 min [20], reactions were stopped by adding DNS (500 μL) and boiling for 15 min. The concentration of reducing sugars was determined spectrophotometrically (Shimadzu UV-1900) by measuring the absorbance at 550 nm and comparing it to a glucose standard calibration curve. Enzyme blanks (50 μL of HrGH45 in buffer A plus 450 μL of buffer A) and substrate blanks (250 μL of 1% *w*/*v* CMC dissolved in buffer A plus 250 μL of buffer A) were included and treated similarly. Assays were performed in triplicate. One unit (U) of cellulase activity was defined as the amount of the enzyme that produces reducing sugars equivalent to 1 μmol of glucose per min under the conditions described above. The specific activity was defined as U/mg of protein.

Various substrates, including filter paper Whatman 1 (50 mg, 1 × 6 cm, GE Healthcare), 1% (*w*/*v*) microcrystalline cellulose (High Purity), and 1% (*w*/*v*) commercial cotton, were used to examine the capacity of HrGH45 to hydrolyze different types of cellulose. As described above, the assays were slightly modified: filter paper samples were incubated for 60 min while microcrystalline cellulose and cotton samples were for 24 h [19, 20]. Enzyme and substrate blanks were included and treated similarly. Assays were performed in triplicate. A commercial cellulase from *Aspergillus niger* (Sigma-Aldrich) was used as a positive control.

We determined the cellobiohydrolase and *β*-glucosidase activities of HrGH45 by measuring the contents of *p*-nitrophenol released from the hydrolysis of *p*-nitrophenyl-*β*-D-cellobioside (*p*NPC, Sigma-Aldrich) and *p*-nitrophenyl-*β*-D-glucopyranoside (*p*NPG, Sigma-Aldrich), respectively, as described by Deshpande *et al*., [21]. The reaction mixture contained 250 μL of 5 mM *p*NPC or *p*NPG dissolved in buffer A and 250 μL of HrGH45 (0.1 mg/mL) in buffer A. After incubation at 45°C for 30 min, reactions were stopped by adding 1 M Na_2_CO_3_ (500 μL). The amount of *p*-nitrophenol released was calculated from its molar extinction coefficient of 18,500 M^-1^cm^-1^ at 410 nm. Substrate blanks (250 μL of 5 mM *p*NPC or *p*NPG dissolved in buffer A and 250 μL of buffer A) were included and treated similarly. Assays were performed in triplicate. One unit (U) of cellulase activity was defined as the amount of the enzyme that produces 1 μmol of *p*-nitrophenol per min under the above conditions. The specific activity was defined as U/mg of protein.

To investigate the substrate-binding mode of HrGH45, we used cellobiose (G2), cellotriose (G3), cellotetraose (G4), and cellopentaose (G5) as substrates. Each cello-oligosaccharide was incubated with the enzyme and their hydrolysis products were subsequently analyzed by thin layer chromatography (TLC). The reaction mixture consisted of 500 μL of 2 mg/mL cello-oligomer dissolved in buffer A and 500 μL of HrGH45 (0.1 mg/mL) in buffer A. After incubation at 45°C for 24 h [22], reactions were stopped by boiling for 10 min. Ten μL of each sample were mixed with ten μL of ethanol (EtOH) [23], then spotted onto the TLC plate (Macherey-Nagel, ALUGRAM Xtra SIL G/UV_254_ Aluminum Sheets) each 0.5 μL with a micropipette. Substrate blanks (500 μL of 2 mg/mL cello-oligosaccharide dissolved in buffer A and 500 μL of buffer A) were included and treated similarly. TLC was developed at 25°C using the double-ascending method and a mixture of ethyl acetate/acetic acid/distilled water (AcOEt/AcOH/H_2_O) in a volume ratio of 3:2:1 as the mobile phase. After separation, the TLC plate was air-dried, sprayed with 10% (*v*/*v*) H_2_SO_4_ in EtOH, and heated to 120°C until the resolved products were visualized [22]. Glucose (G1) and cello-oligomers G2–G5 (Sigma-Aldrich) were used as standards.

### MALDI-TOF mass spectrometry (MALDI-TOF MS)

HrGH45 was concentrated by precipitation with EtOH as follows: four volumes of EtOH were added to one volume of the enzyme solution, they were vortexed and kept at -55°C for 60 min. The mixture was then centrifugated (13,000 rpm for 15 min at 4°C) and the supernatant was discarded. The pellet was vacuum-dried and resuspended with Milli-Q water (10 μL). The sample was placed onto the MALDI plate in a 1:5 sample-matrix ratio and then was air-dried at 25°C. The matrix used was a saturated solution of Super-DHB (Sigma-Aldrich) dissolved in 0.1% (*v*/*v*) TFA and 30% (*v*/*v*) ACN in H_2_O. A Microflex MALDI-TOF mass spectrometer (Bruker Daltonics) and the FlexAnalysis 3.0 software (Bruker Daltonics) were used for mass spectra recording and peak detection. Before the acquisition, the spectrometer was calibrated using thaumatin (22.2 kDa), glucose isomerase (43.25 kDa), and BSA (66.6 kDa). Spectrum was recorded in positive ion linear mode.

### Molecular mass and behavior in solution

The molecular mass and association state of native HrGH45 in solution were determined after a gel filtration column was calibrated. A freshly purified cellulase solution (0.1 mg/mL in buffer A) was loaded onto an Agilent Bio SEC-3 HPLC column (3 μm, 150 Å, 7.8 I.D. × 150 mm, Agilent Technologies) installed in an Agilent 1100 HPLC instrument (Agilent Technologies). Chromatography was developed at a 1 mL/min flow rate using buffer A as eluent. Protein standards used for calibration were tetrameric glucose isomerase (173 kDa), BSA (66.6 kDa), Agave chitinase (31.9 kDa), thaumatin (22.2 kDa), and lysozyme (14 kDa).

### Zymography

In-gel assays for cellulase activity were carried out as detailed by Cano-Ramírez *et al*. [24] and Champasri *et al*. [25] with slight modifications: HrGH45 was loaded onto a 4% stacking gel without any reducing agents or heating. Moreover, CMC was incorporated into the 12% resolving phase gel at a final concentration of 0.1% (*w*/*v*). After electrophoresis, the gel was immersed for 30 min in 5% (*v*/*v*) Triton X-100 (Sigma-Aldrich) in buffer A, washed with Milli-Q water, and incubated overnight at 37°C in buffer A. The gel was then stained with 0.2% (*w*/*v*) Congo red (Sigma-Aldrich) in EtOH for 60 min and destained with 1.5 M NaCl until clear-translucent bands were observed. A second identically prepared gel was stained with Coomassie brilliant blue R-250 after electrophoresis.

### Glycosylation detection

According to the periodic acid-Schiff reagent method, glycoproteins on a 12% SDS-PAGE gel were stained using the Pierce Glycoprotein Staining Kit (Thermo-Fisher Scientific) following the manufacturer’s protocol. The phenol-sulfuric acid method [26] was employed to estimate total carbohydrates. Glucose was used as a standard. Assays were performed in triplicate.

### Effects of pH and temperature on enzyme activity

pH dependence was analyzed at 45°C in reaction mixtures adjusted to pH 3.0–10.0 using 40 mM Britton-Robinson buffer. Temperature dependence was examined in 40 mM Britton-Robinson buffer pH 7.0 from 4 to 75°C. Enzyme activity was determined as detailed in *Enzyme assays*: *cellulase activity*, *specificity*, *and mode of action*. Experiments were performed in triplicate.

### Circular dichroism

Circular dichroism (CD) spectroscopy was used to estimate the contents of secondary structure elements and folding properties of HrGH45. CD measurements were acquired in the far-UV region (190–260 nm) using a J-1500 spectropolarimeter (JASCO) equipped with a Peltier device as a temperature control system. A cellulase solution (0.2 mg/mL in buffer A) was dialyzed (Spectrum Spectra/Por 3,500 Da MWCO) against distilled water and then filtered (Millipore, Durapore PVDF Membrane Filter, Ø 0.22 μm). Experiments were performed at 25°C using a 1-mm path-length quartz cuvette and the following spectral acquisition parameters: response time, 1 s; bandwidth, 1 nm; scanning rate, 10 nm/min. CD data were corrected from solvent contributions. Three scans were averaged to obtain the enzyme’s final spectrum. The latter was presented in terms of mean residue molar ellipticity ([θ] deg∙cm^2^∙dmol^-1^), which was calculated using a mean residue weight of 110 g/mol [27]. The BeStSel server [28, 29] analyzed the final CD spectrum.

### Peptide mass fingerprinting

To obtain information on the primary structure of HrGH45, it was in-gel digested using the Protease Profiler Kit (Sigma-Aldrich) according to the manufacturer’s instructions. Briefly, after electrophoresis, stained protein bands were carefully excised from the gel, distained (250 mM NH_4_HCO_3_ in 50% *v*/*v* ACN), reduced (50 mM tris-(2-carboxyethyl)phosphine, 10 min at 60°C), alkylated (100 mM iodoacetamide, 60 min at 25°C in the dark), dehydrated (ACN), and subjected to proteolytic cleavage reactions, which were performed with 0.4 μg of protease in 120 μL of 40 mM NH_4_HCO_3_ in 9% (*v*/*v*) ACN at 30°C for 20 h. The peptides formed were extracted and vacuum dried. They were then dissolved with 10 μL of the matrix solution and placed onto the MALDI plate. The matrix used was a saturated solution of *α*-CHCA (Sigma-Aldrich) dissolved in 0.1% (*v*/*v*) TFA and 30% (*v*/*v*) ACN in H_2_O. A Microflex MALDI-TOF mass spectrometer (Bruker Daltonics) and the FlexAnalysis 3.0 software (Bruker Daltonics) were used for mass spectra recording and peak detection. Before the acquisition, the spectrometer was calibrated using “Tube 4: Peptide Calibration Standard” from the Starter Kit for MALDI-TOF MS (Bruker Daltonics). Spectra were recorded in positive ion reflector mode. For protein identification, the UniProt databases were used. The analysis was performed using MASCOT 2.4 (Matrix Science) installed on a local server assuming the following parameters: fixed modification, carbamidomethylation (C); variable modification, oxidation (M); mass tolerance, 0.1%; missed cleavages, up to 2.

### Identification of HrGH45 gene

We employed a bioinformatic approach to identify the HrGH45 gene. The peptides formed during protein sequencing experiments were identical to the internal sequence of an endo-1,4-*β*-D-glucanase from the disc abalone *Haliotis discus discus* (UniProt B6RB06). Thus, based on the disc abalone cellulase mRNA (GenBank EF103350), we searched for a similar sequence in the red abalone genome (BioProject PRJNA434455, BioSample SAMN08558906P). The *H*. *rufescens* whole genome shotgun project has the accession entry QGMO00000000. We used the project QGMO01000000, which consists of sequences QGMO01000001-QGMO01008371. The HrGH45 gene was found in the sequence QGMO01000012.

### Structural modeling of HrGH45 and docking with *β*-cellopentaose

We used the artificial intelligence program AlphaFold [30] to obtain a 3D model of HrGH45 based on its amino acid sequence, here elucidated. Once the structure was built, we employed Coot 0.9.8.8 [31] to correct geometrical parameters. The overall stereochemical quality of the model was assessed using PROCHECK [32]. Molecular docking determinations were performed using AUTODOCK 4.2.1 [33] and *β*-cellopentaose with the anomeric center at the reducing end with *β*-configuration. Initially, potential binding sites were detected based on a blind docking of a box sufficiently large to contain the whole enzyme (110 Å × 110 Å × 110 Å), centered at the protein center. We performed then a focused docking with a smaller box (55 Å × 55 Å × 55 Å), centered according to the best energy result obtained from the blind docking. Using the AlphaFold-Multimer program [34], we determined the possible trimeric association model of HrGH45.

## Results

### Purification of HrGH45

**Fig 1:**
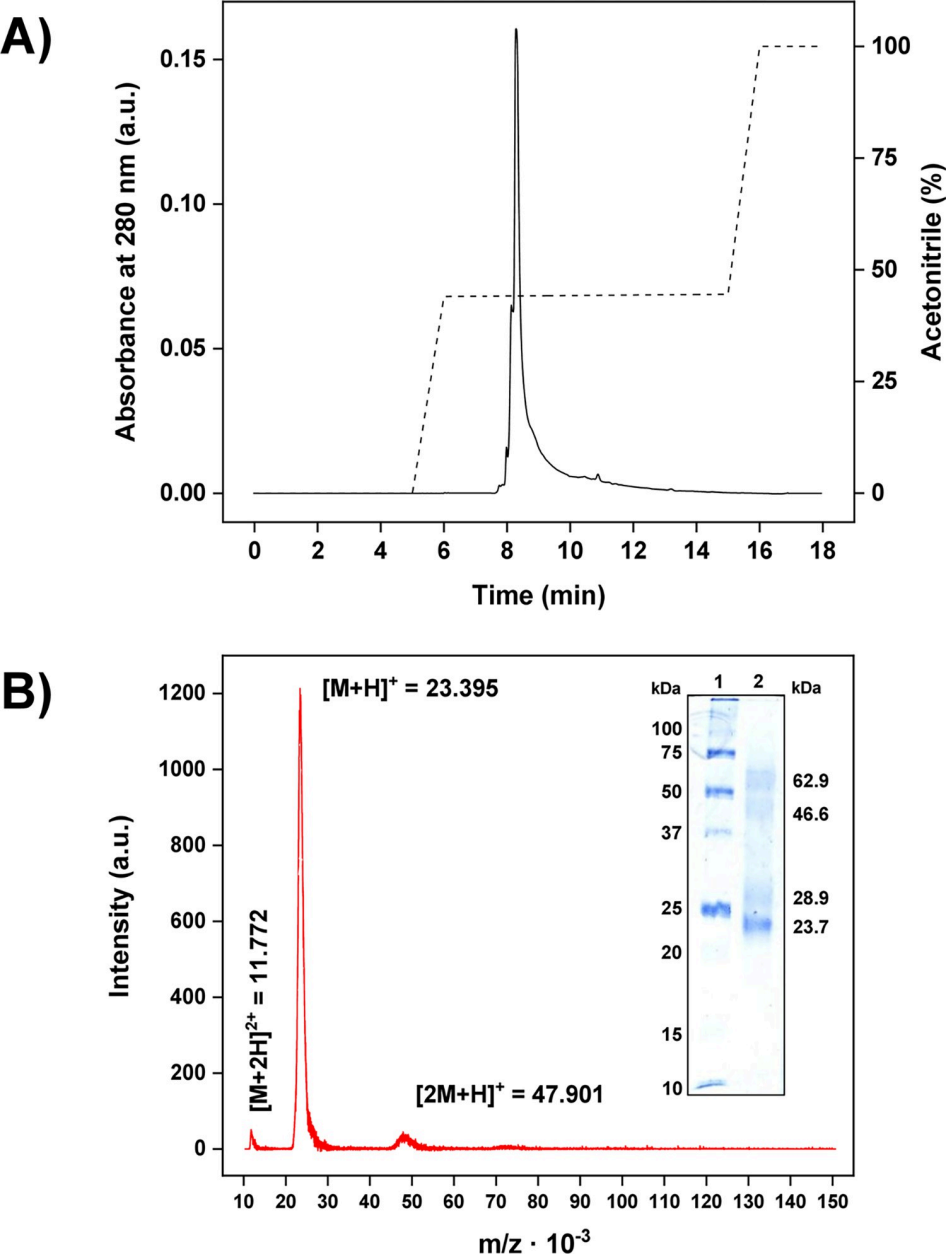
Homogeneity of HrGH45. **(A)** C_18_-reversed-phase HPLC elution profile of HrGH45. **(B)** MALDI-TOF mass spectrum of HrGH45. The doubly charged ion ([M+2H]^2+^ = 11.772), the molecular ion ([M+H]^+^ = 23.395), and a protein aggregate ([2M+H]^+^ = 47.901) were detected. The inset shows the SDS-PAGE pattern of HrGH45. 1, Precision plus protein unstained standards; 2, HrGH45.

Endoglucanase (endo-1,4-*β*-D-glucanase, EC 3.2.1.4) was isolated from the hepatopancreas of *H*. *rufescens* by ammonium sulfate precipitation and two chromatographic methods. The primary purification step was achieved by size-exclusion chromatography. HrGH45 was purified at a yield of 2% with a specific activity toward 0.5% (*w*/*v*) CMC of 53.5 U/mg. The purity and homogeneity of HrGH45 were determined using C_18_-reversed-phase chromatography, where a single, sharp peak was observed in the fraction eluted with 44% (*v/v*) ACN (Fig 1A). Nonetheless, during standard SDS-PAGE, HrGH45 appeared as four protein bands with estimated molecular masses of 23.7, 28.9, 46.6, and 62.9 kDa (Fig 1B, *inset*). These masses were confirmed by MALDI-TOF MS, where mainly two bands were acquired; the base peak around *m*/*z* = 23.395 kDa, approximately 23.4 kDa, and the dimer ([2M+H]^+^ = 47.901 kDa, approximately 47.9 kDa). However, we did not observe a signal around *m*/*z* = 29 kDa (Fig 1B).

### HrGH45 is a glycoprotein and behaves as a trimer

**Fig 2:**
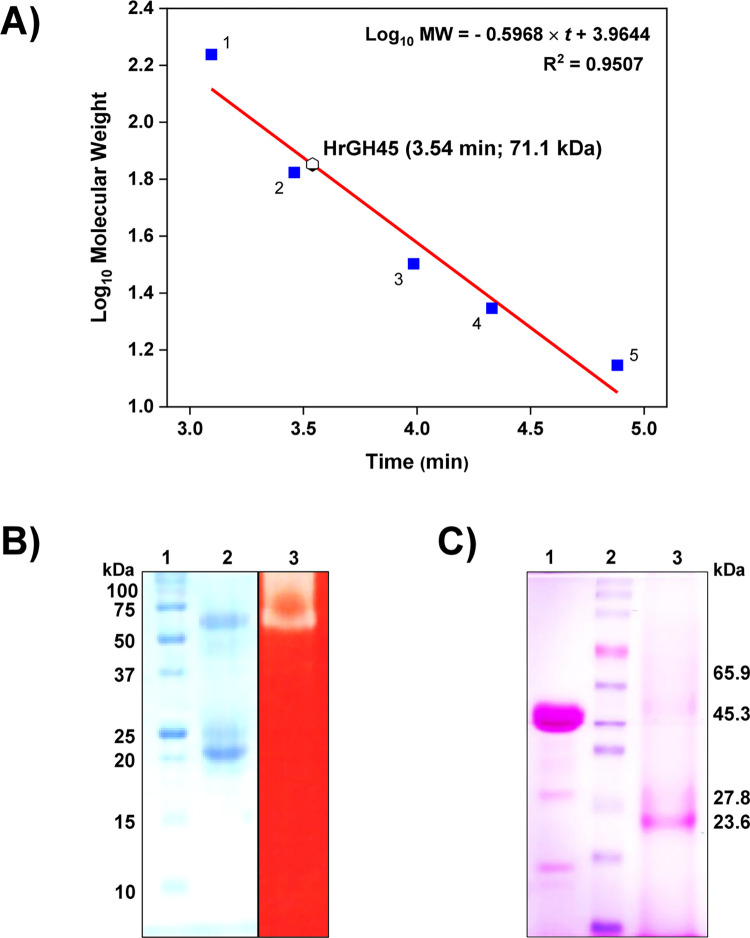
HrGH45 is a glycoprotein and behaves as a trimer. **(A)** Gel filtration calibration curve used to estimate the molecular mass of HrGH45 in aqueous systems. The protein standards used for calibration were: 1, Tetrameric glucose isomerase (173 kDa); 2, BSA (66.6 kDa); 3, Agave chitinase (31.9 kDa); 4, Thaumatin (22.2 kDa); 5, Lysozyme (14 kDa). The solid line represents the calculated calibration curve. **(B)** Zymogram analysis of HrGH45. *Left*: Migration pattern of HrGH45 on 12% SDS-PAGE gel containing 0.1% (*w*/*v*) CMC in the resolving phase. 1, Precision plus protein unstained standards; 2, HrGH45. *Right*: In-gel assay (zymogram) for cellulase activity. 3, HrGH45. Endoglucanase activity is visualized as a clear-translucent band. **(C)** 12% SDS-PAGE gel treated with the Pierce glycoprotein staining kit. 1, Horseradish peroxidase (positive control); 2, Bio Basic prestained protein ladder; 3, HrGH45. Degradation of horseradish peroxidase was evident. Glycoproteins are visualized as magenta bands.

The association state of native HrGH45 in solution was determined by size-exclusion chromatography. Based on a gel-filtration calibration curve (Fig 2A), the estimated molecular mass of HrGH45 was 71.1 kDa. Nevertheless, during MALDI-TOF MS acquisitions, HrGH45 showed a molecular mass of 23.4 kDa. Thus, this biomolecule behaves as a homotrimer in aqueous systems. Besides, active oligomeric forms of HrGH45 on polyacrylamide gels were detected by zymography. After electrophoresis using homogeneous 12% SDS-PAGE gels copolymerized with 0.1% (*w*/*v*) CMC in the resolving phase, Coomassie brilliant blue (Fig 2B *left*) and Congo red (Fig 2B *right*) stains were performed. In these experiments, the migration pattern was like the one previously observed (Fig 1B *inset*). Enzyme activity was detected as a clear-translucent band with a molecular mass of approximately 67 kDa, corresponding to the trimeric conformation of HrGH45 (Fig 2B). No hydrolysis was visualized in the monomeric or dimeric arrangements. This significant observation suggests that HrGH45 functions as a homotrimer.

To determine if HrGH45 was a glycoprotein, we performed a carbohydrate analysis using the phenol-sulfuric acid method [26]. The total sugar content of HrGH45 was 26 ± 3%. Besides, we revealed glycoproteins on a 12% SDS-PAGE gel by the periodic acid-Schiff reagent staining after electrophoresis. This experiment detected four protein bands with estimated molecular masses of 23.6, 27.8, 45.3, and 65.9 kDa (Fig 2C). The migration pattern was like the one previously observed.

### HrGH45 exhibits endo/exo activity and recognizes units of cellopentaose

**Fig 3:**
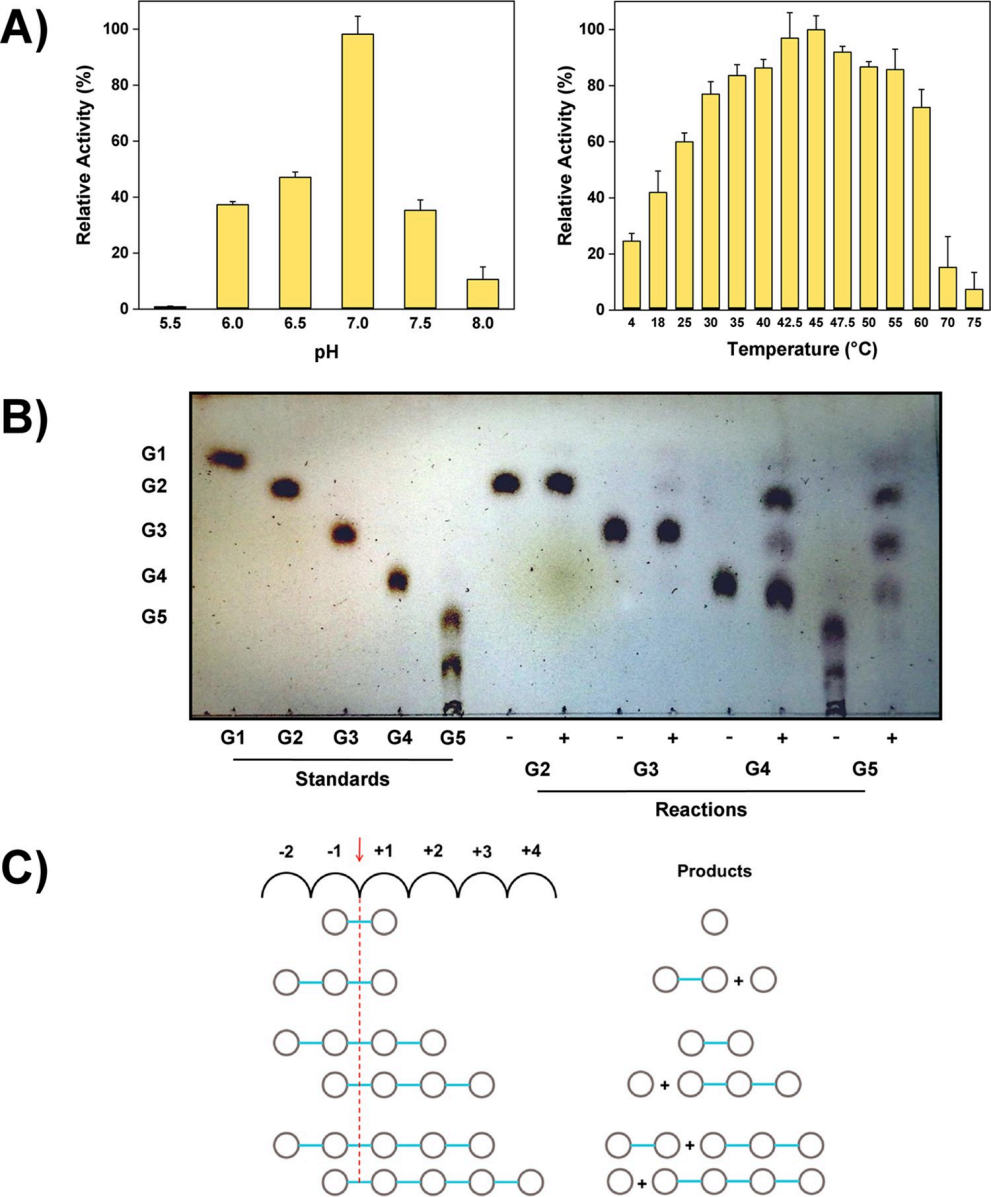
pH and temperature effects on enzyme activity and cellulase mode of action. **(A)** pH and temperature effects. Cellulase activity was determined by measuring the amount of reducing sugars released from the hydrolysis of 0.5% (*w*/*v*) CMC using the DNS method. Assays were carried out in triplicate, and results were expressed as mean ± standard deviation. **(B)** Mode of action of HrGH45: cello-oligosaccharides hydrolysis. TLC was performed using the double-ascending method and a mixture of AcOEt/AcOH/H_2_O (3:2:1) as the mobile phase. After separation, the TLC plate was air-dried, sprayed with 10% (*v*/*v*) H_2_SO_4_ in EtOH, and heated until visualizing the resolved products. +, Hydrolysis reaction; -, Blank reaction; G1, Glucose; G2, Cellobiose; G3, Cellotriose; G4, Cellotetraose; G5, Cellopentaose. **(C)** Hypothesized substrate-binding model of HrGH45. Numbers (-2 to +4) represent the putative sugar-binding subsites. The arrow indicates the cleavage point between subsites -1 and +1. Open circles illustrate D-glucosyl moieties.

HrGH45 showed maximum activity at pH 7.0 and 45°C using 0.5% (*w*/*v*) CMC as substrate in reaction mixtures prepared with 40 mM Britton-Robinson buffer (Fig 3A). Under these conditions, enzyme activity was two times lower than that measured when determined with buffer A at 45°C. For this reason and to compare with other cellulases, the activity measurements were performed using buffer A.

We analyzed the capacity of HrGH45 to hydrolyze different types of cellulose by testing several substrates, including 0.5% (*w*/*v*) CMC, filter paper (50 mg), 1% (*w*/*v*) microcrystalline cellulose, and 1% (*w*/*v*) cotton. The first two were used as models of amorphous cellulose, while the latter as crystalline cellulose. Glycosidase activity was detected on CMC (53.9 ± 1.0 U/mg) and filter paper (10.6 ± 1.2 U/mg). No hydrolysis was observed using microcrystalline cellulose or cotton as substrates. These results confirmed that HrGH45 is a typical endo-1,4-*β*-D-glucanase, suggesting it can hydrolyze amorphous regions in cellulose chains.

The synthetic chromogens *p*NPC and *p*NPG were used as selective substrates for measuring the cellobiohydrolase and *β*-glucosidase activities of HrGH45, respectively. Remarkably, enzyme activity was detected on the agluconic (heterosidic) bond of *p*NPC (6.97 ± 0.17 U/mg) and *p*NPG (1.25 ± 0.01 U/mg), evidencing the cellobiohydrolase type II and *β*-glucosidase activities of HrGH45. Cel III, a blue abalone cellulase [11], showed similar enzymatic properties. However, it should be stressed that the activities of cellobiohydrolase and *β*-glucosidase were lower than those of endoglucanase.

Mechanistic insights into the mode of action of HrGH45 were proposed after identifying, using TLC, the final reaction products of cello-oligosaccharides hydrolysis. HrGH45 slowly cleaved cellobiose and cellotriose to glucose and to cellobiose plus glucose, respectively. Cellotetraose was partially hydrolyzed to cellobiose, and traces of cellotriose plus glucose were also detected. For cellopentaose, the major cleavage took place at its second glycosidic bond, releasing cellobiose plus cellotriose. However, a minor cleavage at its first glycosidic linkage resulted in glucose plus cellotetraose (Fig 3B). The hydrolysis pattern observed on cello-oligomers and the activity detected on chromogenic substrates suggested a subsites arrangement in the HrGH45 active center. As cellopentaose was hydrolyzed entirely, detecting two hydrolysis patterns for this saccharide, we propose that HrGH45 recognizes units of cellopentaose in cellulose chains through six possible substrate-binding subsites and preferentially cleaves at its second glycosidic linkage (Fig 3C).

### HrGH45 is a *β*-sheet rich protein and belongs to the glycosyl-hydrolase family 45

**Fig 4:**
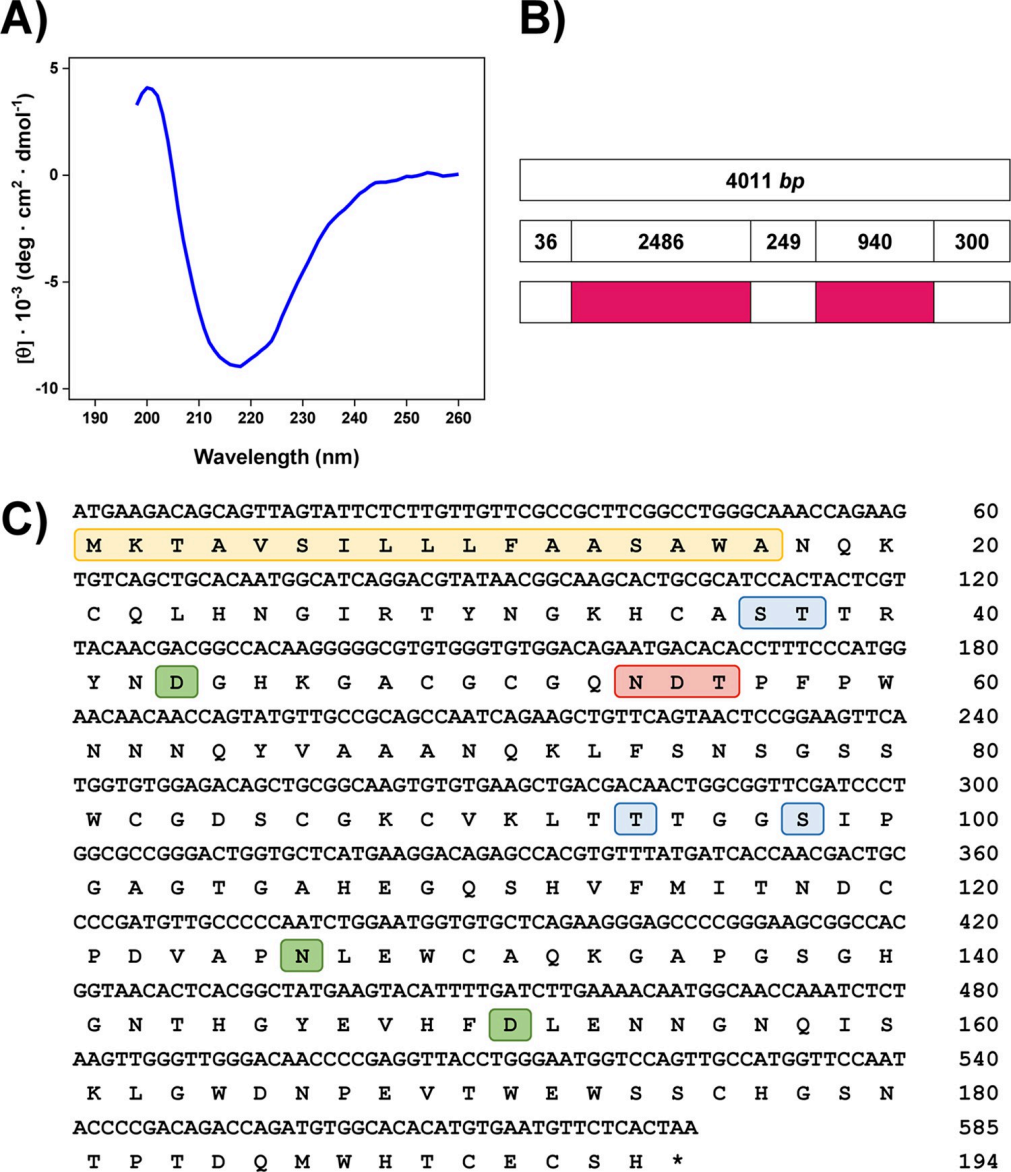
HrGH45 is a *β*-sheet-rich protein and belongs to GH45. **(A)** Far-UV CD spectrum of HrGH45. Ellipticities are reported as mean residue molar ellipticity ([θ] deg∙cm^2^∙dmol^-1^). Three scans were averaged to obtain the final spectrum of HrGH45. CD data were corrected from solvent contributions**. (B**) Schematic representation of HrGH45 gene. White and magenta boxes represent exons and introns, respectively. **(C)** Nucleotide and deduced amino acid sequences of HrGH45. The single-letter amino acid code represents the polypeptide chain below the DNA sequence. Residue numbers for nucleotides and amino acids are indicated to the right of each row. The asterisk denotes the translational stop codon (TAA). In green, catalytic residues; in blue, predicted *O*-glycosylated amino acids; in red, *N*-glycosylation sequon; in yellow, the putative signal peptide for secretion.

Circular dichroism (CD) in the far-UV region was used to analyze the secondary structure and folding properties of HrGH45. The spectrum was characteristic of a *β*-sheet-rich protein with a positive band at 200 nm and a wide negative band centered at 218 nm accompanied by a weak shoulder around 224 nm (Fig 4A). The secondary structure contents of HrGH45, estimated from the CD spectrum using the BeStSel server, were 9.1% *α*-helix, 38.6% *β*-sheet, 11.5% turns, and 40.8% undefined structures. The BeStSel algorithm predicted the structural class, architecture, and topology of HrGH45: all-*β*, *β*-barrel, and Barwin-like endoglucanases, respectively.

HrGH45 was in-gel digested using a battery of different proteases to elucidate its primary structure. Peptides A-C (Table 1), formed after proteolysis with trypsin and Glu-C, were identical to a GH45 endo-1,4-*β*-D-glucanase from the disc abalone (*H*. *discus discus*, UniProt B6RB06). No coincidences were found when analyzing the peptides generated from the hydrolysis with the remaining proteases since they might be decorated with structural glycans or other post-translational modifications, which could shift the peptide molecular masses out of the range for sequencing [35]. We identified the gene encoding HrGH45 in the red abalone genome (PubMed 30657886) [36] and deduced its amino acid sequence. The latter was possible because the disc abalone cellulase mRNA (GenBank EF103350) was used as a reference. The genomic structure of the HrGH45 gene comprised three exons and two introns (Fig 4B). The intron-exon junctions possessed the canonical eukaryotic splice sites (GT-AG). Thus, HrGH45 is biosynthesized by the abalone *per se*.

**Table 1:** Amino acid sequences identified by MALDI-TOF MS in protein sequencing experiments.

Peptide	Start-End	Sequence	[M+H]^+^	Mr	
			Observed	Expected	Calculated
A	34–46	K. HCASTTRYNDGHK. G	1487.686	1486.679	1488.653
B	47–72	K. GACGCGQNDTPFPWNNNQYVAAANQK. L	2769.590	2768.583	2767.187
C	154–168	E. NNGNQISKLGWDNPE. V	1684.816	1683.809	1684.781

Mr, Relative mass. Units are given in Daltons (Da).

The HrGH45 gene encodes a 194 amino acid protein with a theoretical molecular mass of 20.9 kDa. The 17-amino acid region located at the N-terminus of the deduced sequence, M‧K‧T‧A‧V‧S‧I‧L‧L‧L‧F‧A‧A‧S‧A‧W‧A, was predicted as a putative signal peptide by SignalP-5.0 [37]. Accordingly, mature HrGH45 comprised 177 residues with a calculated molecular mass of 19.1 kDa and a theoretical pI of 6.09. This molecular mass was lower than that of 23.4 kDa determined by MALDI-TOF MS. The discrepancy in molecular masses is attributable to glycosylation since HrGH45 is a glycoprotein. Besides, a typical *N*-glycosylation sequon (Asn-Xaa-Thr/Ser, Xaa ≠ Pro) was observed at amino-acid positions of Asn54-Asp55-Thr56. Four putative *O*-glycosylation sites (Ser37, Thr38, Thr94, and Ser98) were identified by NetOGlyc 4.0 [38] (Fig 4C). Twelve cysteine residues were detected in the deduced sequence, and DISULFIND [39] suggested the presence of six disulfide bonds. The consensus amino acid residues of GH45 cellulases, T‧T‧R‧Y‧X‧D [40], were conserved as T38‧T39‧R40‧Y41‧N42‧D43. No carbohydrate-binding module was detected in the deduced sequence.

### The catalytic groove of HrGH45 and its trimeric association

**Fig 5:**
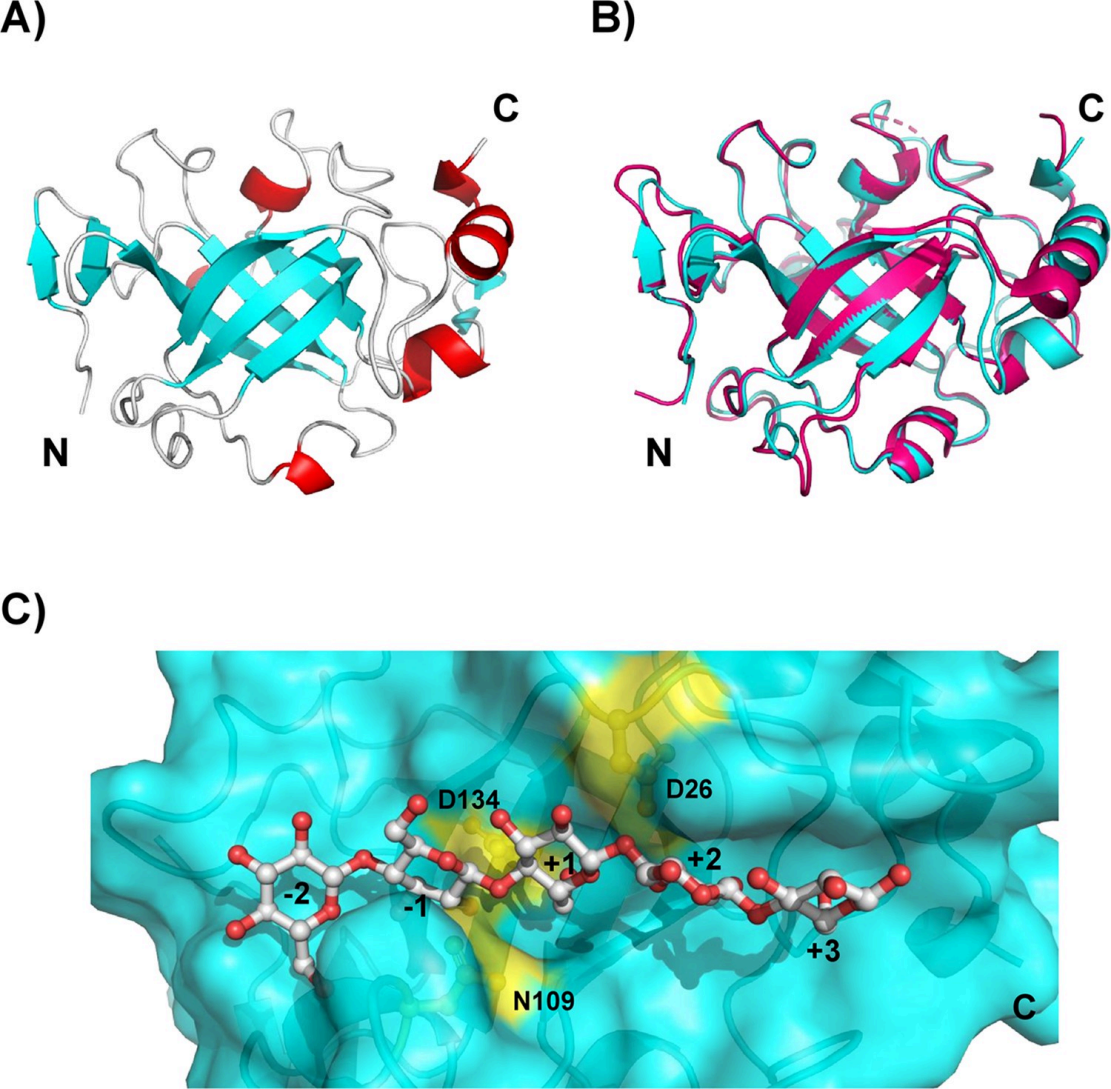
Structure and catalytic groove of HrGH45. **(A)** AlphaFold model for HrGH45. Secondary structure elements are shown in ribbons. **(B)** Superposition of EG27II (PDB 5XC8), a GH45 endoglucanase from the snail *A*.*crosssean* (magenta), and HrGH45 (cyan). **(C)** Surface representation of HrGH45 showing the putative active site residues (yellow) and its possible interaction with cellopentaose.

As our crystallization trials failed, we obtained an AlphaFold model for HrGH45, which exhibits the prototypical six-stranded double-*ψ β*-barrel fold conserved in GH45 endoglucanases. It contains ten *β*-strands, six at the molecule’s core forming the *β*-barrel structure (Fig 5A), as has been elucidated by X-ray crystallography for EG27II, a GH45 endoglucanase belonging to subfamily B from the snail *Ampullaria crossean* (PDB 5XC8) [5]. HrGH45 has a compact and globular structure with dimensions of 35 Å × 40 Å × 50 Å. The twelve cysteine residues present in HrGH45 form six disulfide bridges with the pairings Cys4/Cys18, Cys32/Cys69, Cys34/Cys173, Cys65/Cys175, Cys72/Cys159, and Cys103/Cys113 (without signal peptide), which stabilize the catalytic domain. HrGH45 contains eleven histidine residues, ten of which are situated on the protein’s surface. A short antiparallel *β*-sheet at the N-terminus and two *α*-helices at the C-terminus are formed. A structural alignment of EG27II and HrGH45 showed similar folding (Fig 5B), even though the sequence of amino acids is not fully conserved. The root-mean-square deviation of the C*α* atoms was 0.398 Å. A deep cleft long runs across the protein’s surface near the C-terminus and hosts the catalytically essential residues (Fig 5C).

**Fig 6:**
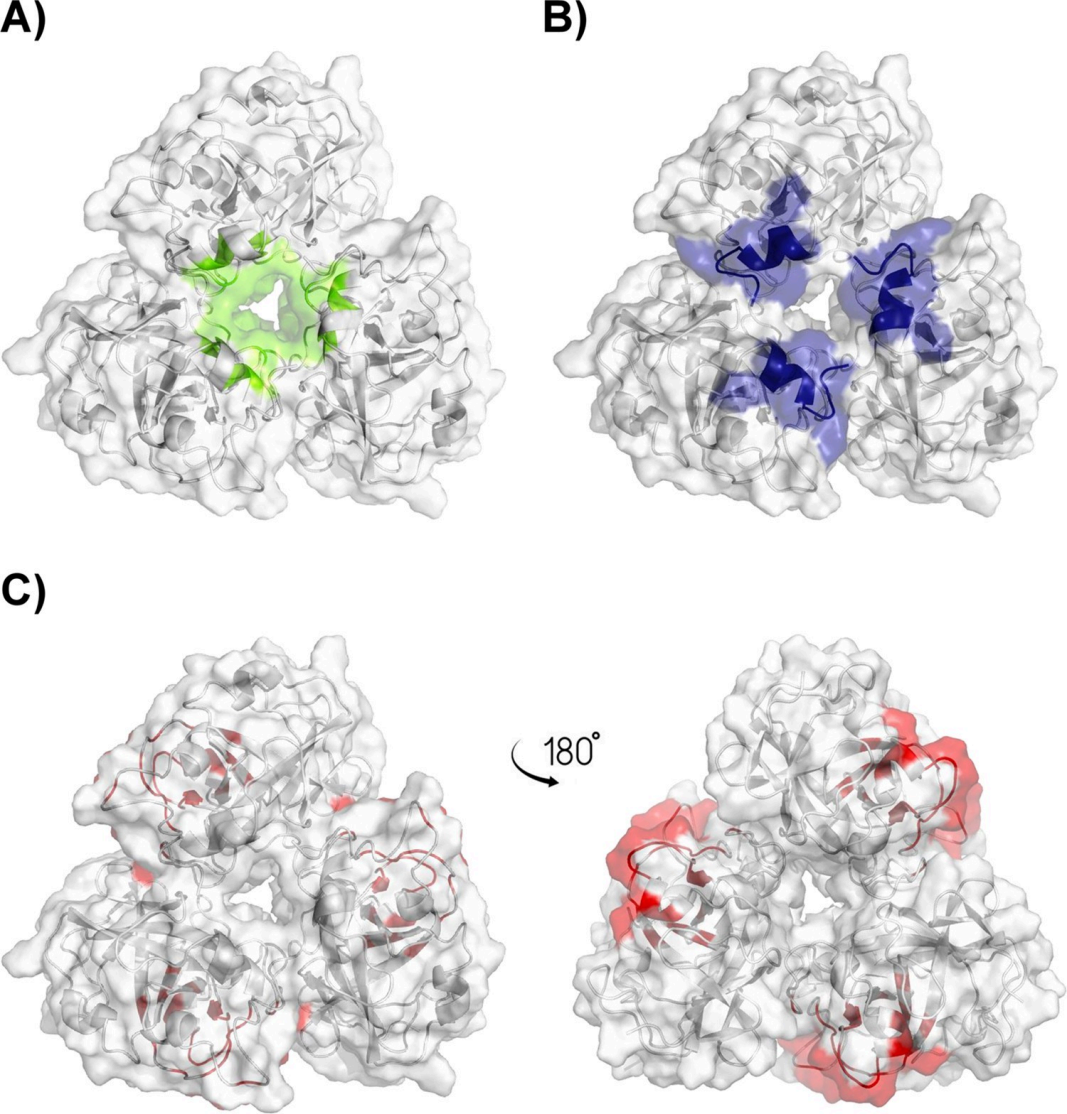
Trimeric organization of HrGH45. Overall view of the AlphaFold-Multimer model for HrGH45 showing **(A)** the buried surface area among monomers (green), **(B)** the putative location of the C-termini (blue), and **(C)** the catalytic clefts exposed to the solvent (red). Monomers are shown in white.

The AlphaFold-Multimer model for HrGH45 showed that three polypeptide chains are associated closely in a triangular mode and interlock to form a trimer with interfaces of 500 Å^2^. In the trimeric organization, a hole runs through the triangle’s center, and the buried surface among monomers constitutes the triangle’s inner edges (Fig 6A). The C-termini come together at the triangle’s front, and the *β*-barrel cores are behind them (Fig 6B). The catalytic clefts are found behind the triangle, on its outer edges, and exposed to the solvent-accessible surface (Fig 6C). The C-terminal region is relevant since some residues in the carboxyl terminus interact with an adjacent subunit. His160 from each monomer contacts His177 from the closest neighbor to stabilize the trimer. Three loops form the interface for the monomer-monomer association in the trimeric assembly. Hydrogen bonds produce the tightest interactions among subunits.

## Discussion

In recent years, microalgae and seaweed have become attractive sources for bioethanol production. However, their exploitation is cost-prohibitive since cellulose is resistant to depolymerization and known soluble cellulases cannot be reused. Thus, new, cheaper, and more sophisticated cellulases are required. In this work, we demonstrated the presence of a trimeric glycosylated endogenous cellulase (HrGH45) in the red abalone hepatopancreas. This discovery follows other reports of GH45 enzymes present in the digestive gland of marine mollusks, such as the sea hare *Aplysia kurodai* [1], the snail *A*. *crossean* [5], and the blue mussel *Mytilus edulis* [41]. We studied the red abalone cellulase, focusing on its oligomeric state and structural folding, comparing it with a related GH45 endoglucanase from the snail *A*. *crossean* (EG27II), which belongs to subfamily B [5].

HrGH45 was purified at a yield of 2% with a specific activity toward 0.5% (*w*/*v*) CMC of 53.5 U/mg. These values were similar to those informed for AkEG21, a GH45 cellulase from the sea hare *A*. *kurodai*, with 3.3% yield and 67.3 U/mg [22]. Based on the purity and homogeneity results obtained for HrGH45, we suggest that protein bands on SDS-PAGE at 46.6 and 62.9 kDa are oligomers since it has been reported that SDS may induce protein oligomerization [42, 43]. Moreover, it is becoming increasingly clear that several types of proteins, like glyco- and phosphoproteins, may exhibit an anomalous electrophoretic behavior on standard SDS-PAGE due to abnormal binding of SDS or increase the SDS-resistant soluble oligomers [44, 45]. The above could explain the protein band at 28.9 kDa. Likewise, it has also been suggested that many intramolecular disulfide bonds may contribute to unusual behavior of proteins in the presence of denaturants, *i*.*e*., oligomerization by a protein denaturant [46].

Trimeric cellulases have been reported in various organisms and are known for their potential efficiency in cellulose degradation due to increased substrate-binding capacity and cooperativity among subunits [3]. Our results on the association state of HrGH45 differed from others reported for native marine molluscan cellulases, which were considered monomeric enzymes [1, 6, 12, 13, 22, 47–49]. A recombinant endoglucanase of 22.54 kDa belonging to GH45 from the salty water clam, *Corbicula japonica* [7], exhibited similar behavior in zymograms to that found for HrGH45. Furthermore, in dynamic light scattering (DLS) measurements, it was observed that three cellulases from *Haliotis fulgens* [11] were prone to aggregate in solution strongly and that their monomeric forms were inactive. *Sd*Gluc5_26A (PDB 5A8N), a trimeric GH5 enzyme from the marine bacterium *S*. *degradans* that hydrolyses a wide diversity of complex polysaccharides, adopted a trimeric quaternary structure, which was also observed in solution, and the authors proposed that this quaternary arrangement controls the substrate specificity [3]. Notably, our findings suggest that the glycosidase activity of HrGH45 is associated with its trimeric conformation, as demonstrated in the zymogram analysis (Fig 2B). The AlphaFold-Multimer program also predicted the association of HrGH45 as a trimer. The total sugar content of HrGH45 was 26 ± 3%. Cellulases from *Haliotis discus hannai* [12] and *Strongylocentrotus nudus* [50] suggested contents of structural saccharides of 4.2 and 12%, respectively. Moreover, hexoses were quantified in all the purified endoglucanases (16.7, 3.2, 25.2, and 9.9%) and *β*-glucosidases (6.6 and 6.7%) of *A*. *kurodai* cellulolytic system [1]. Our carbohydrate analysis indicated that HrGH45 is a glycoprotein and suggested that this biomolecule is biosynthesized by the abalone *per se*. Additionally, the genomic structure of HrGH45 gene confirmed that *H*. *rufescens* produces this enzyme endogenously. The physiological meaning of glycosylation in marine molluscan cellulases is still obscure; nonetheless, it has been demonstrated that these organisms produce several glycan structures, like those present in mammals, plants, insects, or nematodes [51].

Glycosidase activity was detected using CMC and filter paper as substrates, confirming that HrGH45 is a typical endo-1,4-*β*-D-glucanase. A similar hydrolytic pattern was found when characterizing the GH45 endo-1,4-*β*-D-glucanase from *M*. *edulis* [6] and the glycosyl-hydrolase family 9 (GH9) endocellulases from *Bellamya chinensis laeta* [49] and *H*. *discus hannai* [13]. Furthermore, deficient activity on crystalline cellulose was detected in the GH45 endoglucanases from *Crassostrea rivularis* [47] and *A*. *crossean* [48]. The primary reaction products formed by HrGH45 using cello-oligosaccharides as substrates were closely similar to those reported for the GH9 endoglucanases from the abalone *H*. *discus hannai* and the echinoderm *S*. *nudus* [50]. Likewise, our findings were comparable to the hydrolysis patterns observed for the GH45 endoglucanases from *A*. *kurodai* [1, 22] and *M*. *edulis* [6]. The hydrolysis pattern observed on cello-oligomers and the activity detected on chromogenic substrates confirmed the canonical endo-1,4-*β*-D-glucanase character of HrGH45 and proved its broad substrate specificity.

The contents of secondary structure elements of HrGH45 were comparable to other far-UV CD studies for three *H*. *fulgens* cellulases; however, they exhibited slightly more *α*-helix and *β*-sheet contents [11]. The BeStSel algorithm predicted the topology of HrGH45 into a Barwin-like endoglucanase. Barwin is a plant defense protein from barley seed, and it is structurally similar to the catalytic domain of GH45 endoglucanases [52]. Reciprocally, members of GH45 are structurally related to Barwin [53]. Expansins and lytic transglycosylases, cell wall-modifying enzymes in plants and bacteria, exhibit the typical six-stranded double-*ψ β*-barrel core conserved in GH45 proteins. Although their structures define them as members of GH45, their mode of action is non-hydrolytic [54, 55]. The deduced amino acid sequence of HrGH45 showed high sequence identity with the endo-1,4-*β*-D-glucanases from *H*. *discus discus* (98%, GenBank ABO26608), *A*. *kurodai* (54%, GenBank BAP19116), *C*. *japonica* (53%, GenBank BAH23794), *A*. *crossean* (51%, GenBank ABR92638), and *M*. *edulis* (50%, GenBank CAC59694). Remarkably, they all belong to the glycosyl-hydrolase family 45 subfamily B and lack the presence of a carbohydrate-binding module. As our crystallization trials failed, the AlphaFold model for HrGH45, which exhibited a predicted with high average confidence (pIDDT = 97.19), and a docking simulation of a glucose pentasaccharide in the catalytic cleft of HrGH45 confirmed that Asp26, Asn109, and Asp134 (without signal peptide) constitute the enzyme’s catalytic site. Like other GH45 cellulases belonging to subfamily B, the catalytic acid and catalytic base can be assigned to Asp134 and Asn109, respectively. However, Asp26 is also a critical residue for catalysis [5]. The docking simulation results supported our experimental findings on the mode of action of HrGH45, showing that HrGH45 cleaves the cellopentaose’s second glycosidic bond.

In summary, we isolated and characterized an endogenous glycosylated GH45 endo-1,4-*β*-D-glucanase from the red abalone that exhibits cellulase activity in its trimeric form. This research constitutes an important step in identifying cellulases with possible value-added from abalone industry waste products. Glycosyl-hydrolases in abalone and other marine species are essential in carbohydrate metabolism, facilitating the breakdown and utilization of complex saccharides from their environment and enabling marine organisms to adapt to diverse ecological niches. The study of glycosyl-hydrolases in marine species expands our knowledge of their biochemical diversity but also holds promise for discovering novel enzymes with industrial relevance. Exploration of these remarkable molecular architects in the maritime world underscores the importance of understanding their functions and unlocking their potential for various scientific and applied purposes. Our findings provide valuable insights into cellulases’ functional and structural diversity across different organisms, with potential implications for biotechnological applications and our understanding of cellulose degradation in nature.

## Supporting information

S1 Raw images (PDF)
