# Supplementary material for: A trimeric glycosylated GH45 cellulase from the red abalone (Haliotis rufescens) exhibits endo and exoactivity
Source: PLoS One. 2024 Apr 18;19(4):e0301604. doi: 10.1371/journal.pone.0301604 (PMC11025796; doi:10.1371/journal.pone.0301604)
Supplement: S1 Raw images — (PDF) [file pone.0301604.s001.pdf]

Original images for blots and gels

A trimeric glycosylated GH45 cellulase from the red abalone (*Haliotis rufescens*) exhibits endo and exoactivity

L. Joshua Hernández-Benítez, Miguel A. Ramírez-Rodríguez, Alejandra Hernández-Santoyo, and Adela Rodríguez-Romero

Instituto de Química, Universidad Nacional Autónoma de México, Ciudad de México, México

Gel 1

Gel 1 corresponds to Fig 1B inset in the manuscript

Fig 1B inset: SDS-PAGE pattern of HrGH45

1 2 3 4 5 6 7

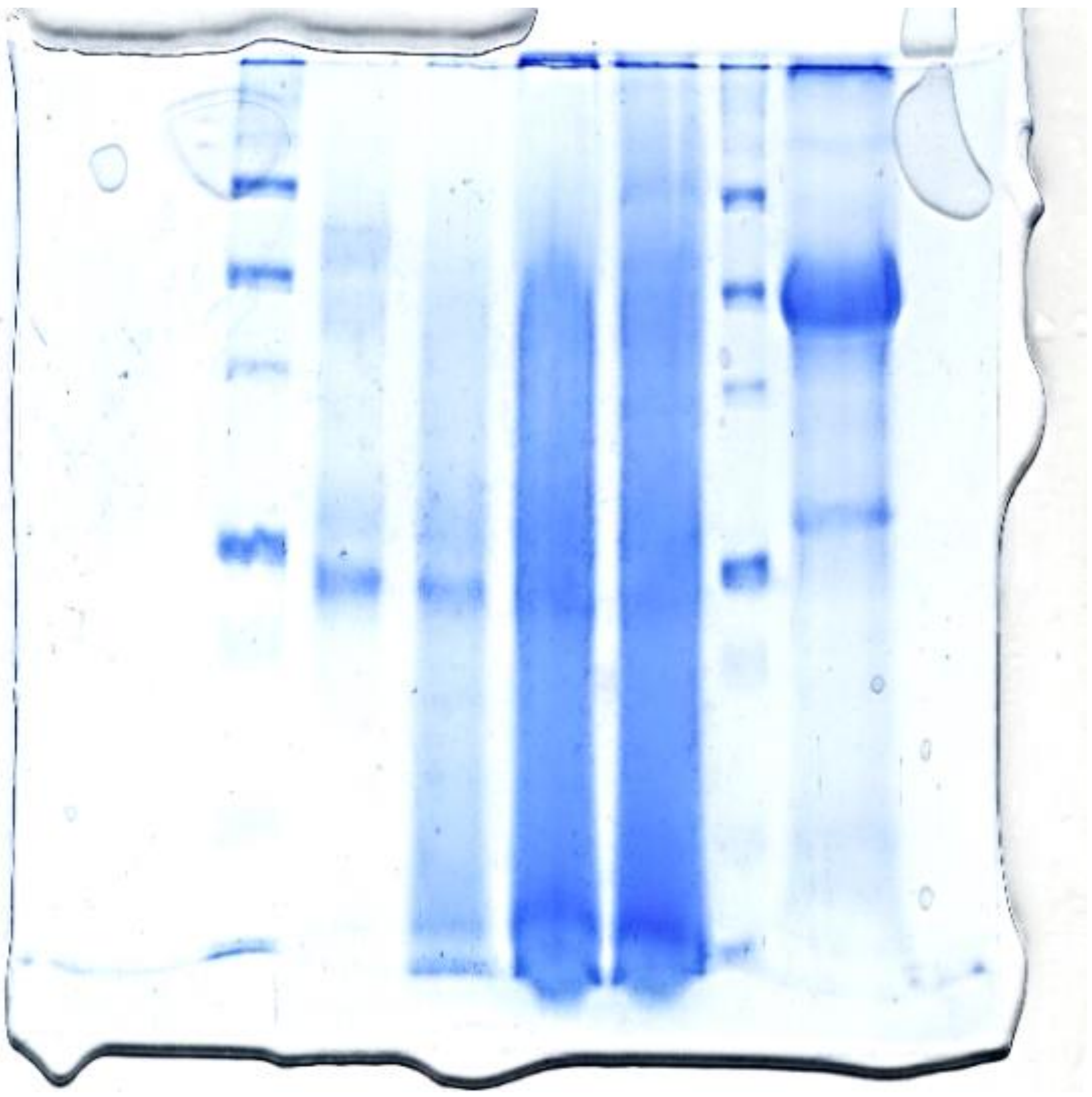

Identity: 1, Precision plus protein unstained standards; 2, HrGH45; 3, Ion exchange chromatography; 4, Ammonium sulfate precipitation (30-60 % saturation); 5, Crude extract; 6, Precision plus protein unstained standards; 7, Recombinant protein from another study.
Method used to capture the image: Photography.
Panel used for publication: Lanes 1-2.

Gel 2

Gel 2 corresponds to Fig 2B Left in the manuscript

Fig. 2B *Left*: Migration pattern of HrGH45 on 12 % SDS-PAGE gel containing 0.1 % (*w*/*v*) CMC in the resolving phase

1 2

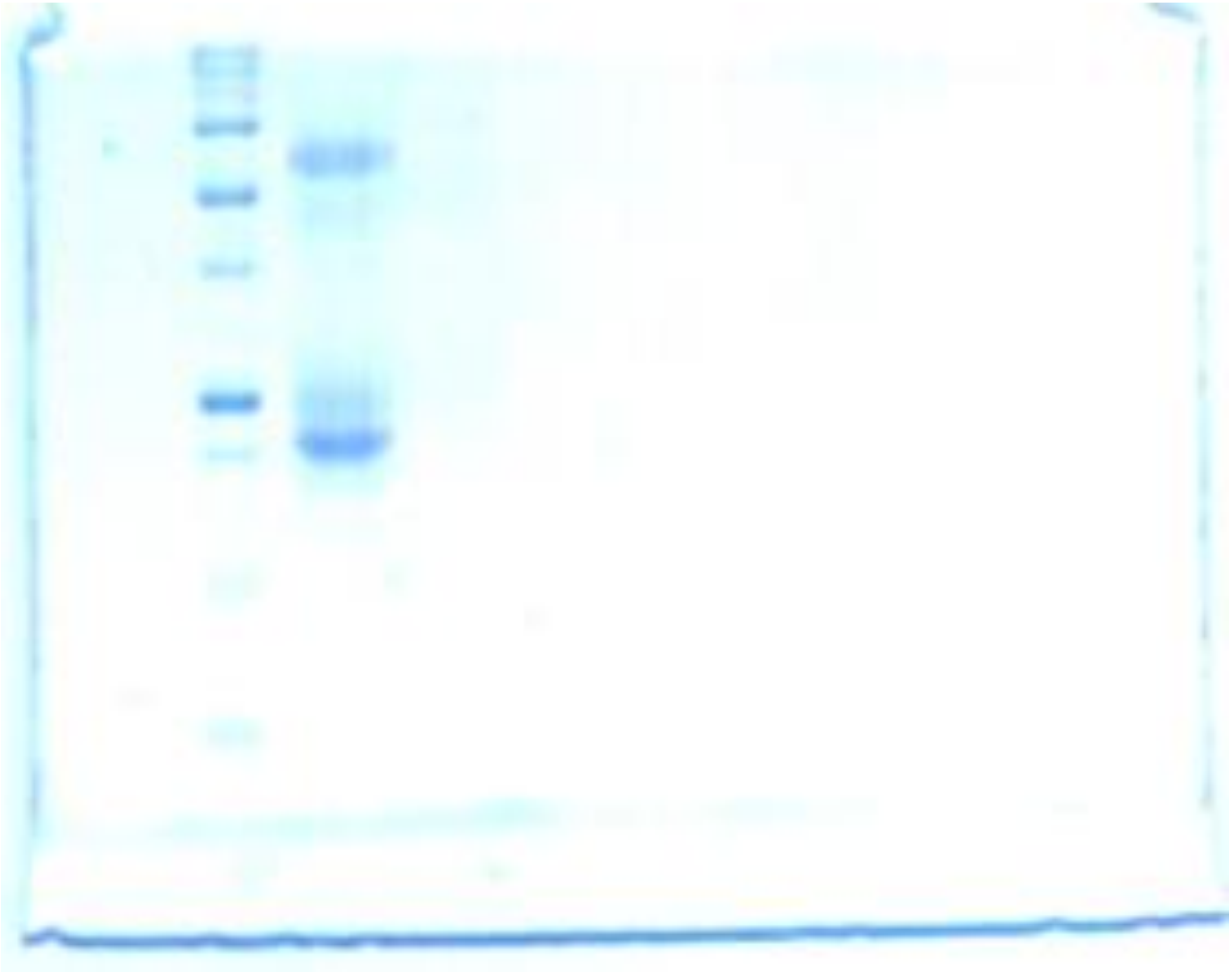

Identity: 1, Precision plus protein unstained standards; 2, HrGH45.
Method used to capture the image: Photography.
Panel used for publication: Lanes 1-2.

Gel 3

Gel 3 corresponds to Fig 2B Right in the manuscript

Fig. 2B *Right*: In-gel assay (zymogram) for cellulase activity.

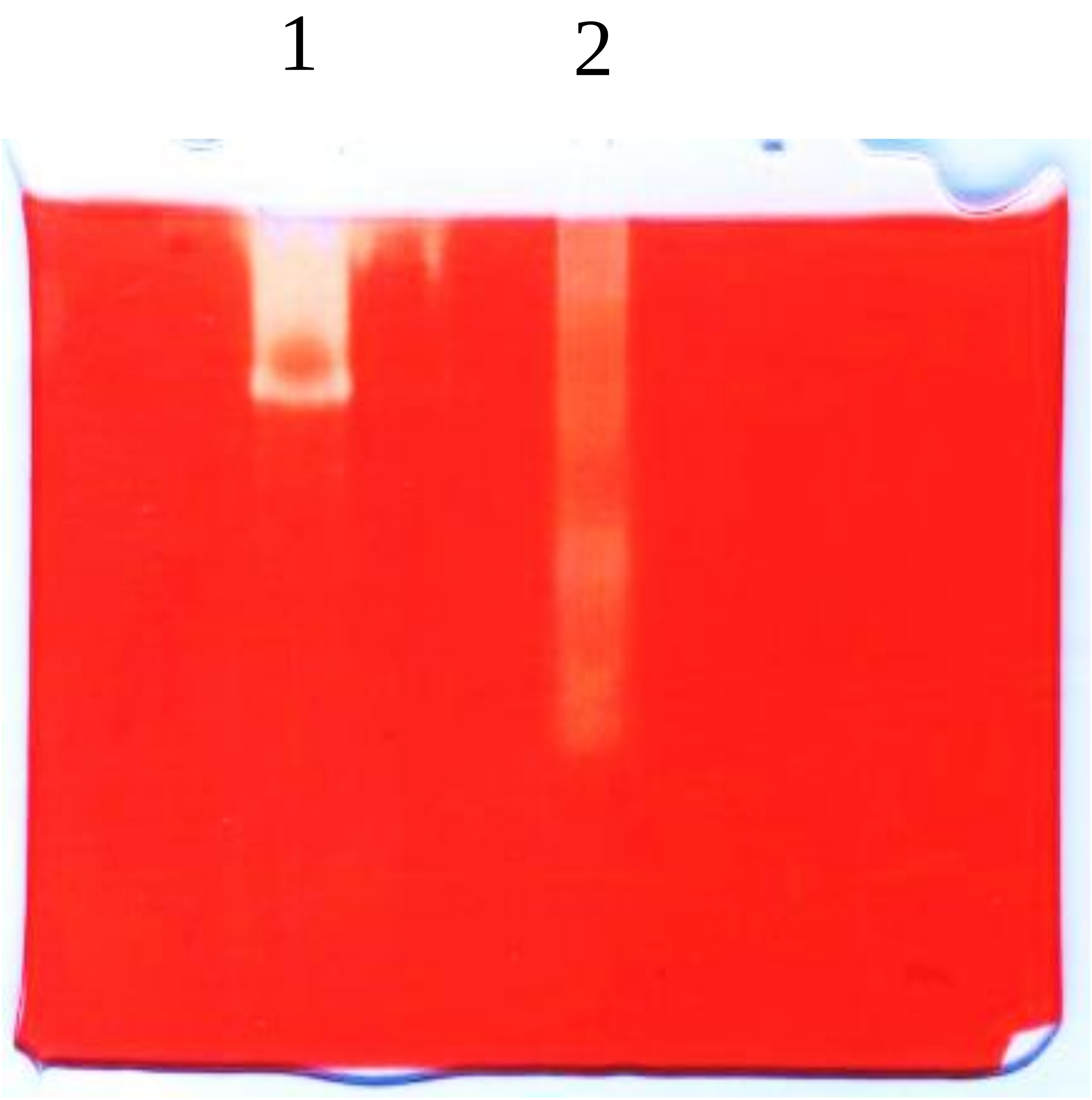

Identity: 1, HrGH45; 2, *Aspergillus niger* cellulase (positive control).
Method used to capture the image: Photography.
Panel used for publication: Lane 1

Gel 4

Gel 4 corresponds to Fig 2C in the manuscript

Fig 2C. 12 % SDS-PAGE gel treated with the Pierce glycoprotein staining kit.

1 2 3 4

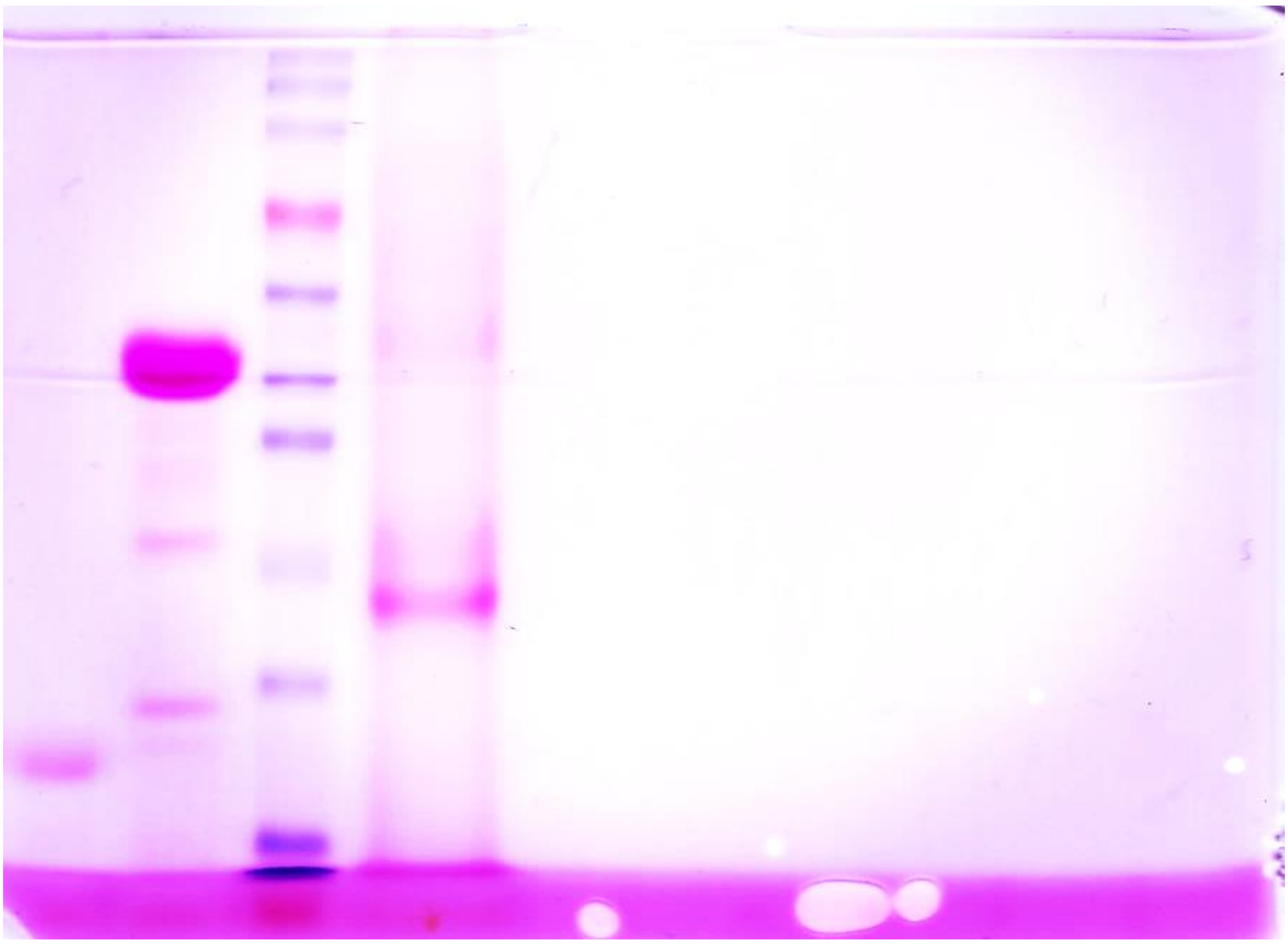

Identity: 1, Glycoprotein from another study; 2, Horseradish peroxidase (positive control); 3, Bio Basic prestained protein ladder; 4, HrGH45.

Method used to capture the image: Photography.

Panel used for publication: Lanes 2-4.
